# Functional and structural analyses of amino acid sequence variation in PDC β-lactamase reveal different mechanistic pathways toward cefiderocol resistance in *Pseudomonas aeruginosa*

**DOI:** 10.1128/aac.00292-25

**Published:** 2025-05-27

**Authors:** Lucía González-Pinto, María Antonia Gomis-Font, Emilio Lence, Michelle Outeda-García, Tania Blanco-Martín, Salud Rodríguez-Pallares, Lucía Sánchez-Peña, Isaac Alonso-García, Juan Carlos Vázquez-Ucha, Alejandro Beceiro, Germán Bou, Concepción González-Bello, Antonio Oliver, Jorge Arca-Suárez

**Affiliations:** 1 Servicio de Microbiología & Instituto de Investigación Biomédica de A Coruña (INIBIC), Complexo Hospitalario Universitario A Coruña 16811, A Coruña, Spain; 2 Servicio de Microbiología & Instituto de Investigación Sanitaria Illes Balears (IdISBa), Hospital Universitario Son Espases 375118, Palma de Mallorca, Spain; 3 CIBER de Enfermedades Infecciosas (CIBERINFEC), Instituto de Salud Carlos III 637284 , Madrid, Spain; 4 Centro Singular de Investigación en Química Biolóxica e Materiais Moleculares (CiQUS), Departamento de Química Orgánica, Universidade de Santiago de Compostela 16780, Santiago de Compostela, Spain; Houston Methodist Hospital and Weill Cornell Medical College, Houston, Texas, USA

**Keywords:** *Pseudomonas aeruginosa*, AmpC β-lactamase, β-lactam resistance, cefiderocol

## Abstract

A wide variety of clinically observed amino acid alterations in the *Pseudomonas aeruginosa* chromosomal β-lactamase AmpC (*Pseudomonas*-derived cephalosporinase [PDC]) are associated with increased resistance to cefepime, ceftolozane/tazobactam, or ceftazidime/avibactam, but their impact on cefiderocol resistance is unclear. We took advantage of a previously engineered collection of wild-type (PAO1) and iron uptake-deficient (PAO Δ*piuC*) *P. aeruginosa* isolates producing 19 distinct PDC variants with substitutions in key catalytic regions. While most variants had moderate effects on cefiderocol minimum inhibitory concentrations compared to PDC-1, the E219K (Ω-loop) and L293P (helix H10) variants significantly affected cefiderocol activity. Kinetic studies revealed that both mutations improve cefiderocol hydrolysis through different enzymatic mechanisms compared to PDC-1 (*K*_m_ = 85.29 µM, *k*_cat_ = 0.0036 s^−1^, and *k*_cat_/*K*_m_ = 0.00004 µM^−1^ s^−1^), leading to enhanced turnover in PDC E219K (*K*_m_ = 465.64 µM, *k*_cat_ = 0.45 s^−1^, and *k*_cat_/*K*_m_ = 0.00096 µM^−1^ s^−1^) and improved affinity in PDC L293P (*K*_m_ = 2.69 µM, *k*_cat_ = 0.0036 s^−1^, and *k*_cat_/*K*_m_ = 0.00135 µM^−1^ s^−1^). These mechanisms are also involved in resistance to ceftolozane and cefepime, identified as the preferred substrates for the E219K and L293P variants, respectively. Molecular dynamics (MD) simulation studies revealed that (i) rigidification of the Ω-loop in PDC E219K promotes optimal accommodation of the R^1^ group of cefiderocol, enhancing nucleophilic attack by the catalytic serine; (ii) the less folded conformation of helix H10 in PDC L293P improves cefiderocol accommodation in the active site by establishing stronger hydrogen-bonding interactions with the R^2^ group. Our findings demonstrate that the PDC β-lactamase may take advantage of the structural similarities between cefiderocol and other cephalosporins and accelerate hydrolysis by accommodating the E219K or L293P amino acid replacements.

## INTRODUCTION

The clinical development of new β-lactam antibiotics to treat infections caused by *Pseudomonas aeruginosa* represents a major challenge regarding Gram-negative pathogens (1). The ability of *P. aeruginosa* to import broad-spectrum resistance mechanisms through horizontal gene transfer (e.g., extended-spectrum β-lactamases [ESBLs] or metallo-β-lactamases) is of growing concern, as it reduces the efficacy of most β-lactam antibiotics and β-lactamase inhibitors, particularly against high-risk clones, which are particularly prone to acquire broad-spectrum β-lactamases. However, the main challenge for new antipseudomonal treatments once they become clinically available is to limit the development of resistance through mutational mechanisms (2). The stepwise accumulation in the same strain of mutations leading to upregulation of *ampC* and efflux operons, in combination with OprD inactivation, commonly results in difficult-to-treat phenotypes (3). New β-lactam/β-lactamase inhibitor combinations with increased stability against these classic *P. aeruginosa* β-lactam resistance mechanisms have recently become available, namely, ceftolozane/tazobactam, ceftazidime/avibactam, and imipenem/relebactam (4). However, since their commercialization, strains with more complex and refined resistance mechanisms are emerging. The development of resistance to ceftolozane/tazobactam or ceftazidime/avibactam commonly involves the rearrangement of key motifs involved in the catalytic cycle of the intrinsic AmpC β-lactamase (designated *Pseudomonas*-derived cephalosporinase [PDC]), rendering both combinations ineffective (5, 6). On the other hand, the selection of mutations leading to upregulated efflux in isolates carrying baseline inactivating mutations in OprD commonly increases the minimum inhibitory concentration (MIC) of imipenem/relebactam above resistance breakpoints (7). In this context, the arsenal of available antipseudomonal agents must be expanded.

Cefiderocol is the most recently Food and Drug Administration-approved β-lactam antibiotic with antipseudomonal activity (8). This newly developed agent incorporates structural motifs from earlier cephalosporins, such as the carboxypropanoxymino group of ceftazidime or ceftolozane on the R^1^ substituent and the pyrrolidinium group of cefepime in position C3 (Fig. S1A). These structural changes provide increased stability against intrinsic (PDC) or horizontally acquired β-lactamases (including metallo-β-lactamases, such as VIM and IMP types) and also enhanced PBP binding affinity. However, the most distinctive attribute of cefiderocol is probably the presence of a chlorocatechol moiety in the R^2^ group, which enables cefiderocol to bind free iron (Fe^3+^) and penetrate the periplasmic space through siderophore-mediated pathways at higher rates than any other β-lactam antibiotic (via the so-called “Trojan horse” mechanism) (9, 10). These combined features result in a compound with high *in vitro* activity against most priority Gram-negative pathogens, including *P. aeruginosa*, regardless of the underlying mechanism of resistance (11). However, widespread β-lactamases acquired by horizontal gene transfer and with high activity toward broad-spectrum cephalosporins, such as NDM-1 or extended-spectrum oxacillinases (e.g., OXA-14 and OXA-15), are increasingly encountered in cefiderocol-resistant *P. aeruginosa* strains. These β-lactamases are damaging the potential of cefiderocol as one of the most promising antipseudomonals in recent years (12, 13). Moreover, analysis of libraries of transposon mutants and large collections of clinical strains has demonstrated that the functional loss of genes related to iron uptake, such as *pirA*, *pirR*, *piuA* and *piuC*, is another major driver of cefiderocol resistance in *P. aeruginosa* (14). Beyond carbapenemases, ESBLs, and iron transport systems, another resistance mechanism that may also affect the activity of cefiderocol but that has been studied in far less detail is the modification of the intrinsic *P. aeruginosa* PDC β-lactamase. Due to the high-sequence polymorphism of this enzyme, more than 600 amino acid variants have been described to date (15). While most of these changes have little or no impact on the natural hydrolytic substrate profile of the PDC enzyme, specific rearrangements in catalytically important regions can have significant therapeutic implications. This has been exemplified by clinical variants found in strains with increased resistance to cefepime or the combinations ceftolozane/tazobactam and ceftazidime/avibactam (16).

However, the mechanisms by which these variant enzymes differentially accelerate cefiderocol hydrolysis remain unclear. Building on the knowledge gained in our previous studies on PDC β-lactamase variants and their role in resistance to ceftolozane/tazobactam, ceftazidime/avibactam, and cefiderocol, increasingly reported in the clinical setting, we sought to understand the impact of specific amino acid substitutions on the enzymatic mechanism of PDC, particularly focusing on cefiderocol resistance. By taking advantage of a previously engineered panel of wild-type and iron uptake-deficient *P. aeruginosa* recombinant isolates enriched with up to 19 different PDC variants harboring substitutions in key catalytic regions (17), we were able to demonstrate the differential effect of the E219K and L293P substitutions on cefiderocol resistance. Moreover, by using kinetic assays and molecular dynamics simulation studies, we have elucidated how these substitutions affect cefiderocol hydrolysis through different enzymatic pathways and lead to increased turnover in the E219K variant and optimized cefiderocol binding affinity in the L293P variant, mechanisms shared with ceftolozane and cefepime, respectively. Our findings are of particular importance, as they contribute to predicting and anticipating potential strategies to overcome cefiderocol resistance in *P. aeruginosa*, which poses a current major clinical challenge.

## RESULTS

### Different effects of PDC amino acid substitutions on cefiderocol resistance

To determine the role of PDC amino acid substitutions in cephalosporin resistance, we assessed the activity of ceftazidime, ceftazidime/avibactam, ceftolozane/tazobactam, cefepime, and cefiderocol against 20 PAO1 and 20 PAO Δ*piuC* transformants producing the parental PDC-1 and 19 different variants. Comparative MIC data for the transformants are summarized in Table 1. For the PAO Δ*piuC* transformants, only cefiderocol MICs are shown, as *piuC* inactivation had a negligible effect on the MICs of the other β-lactam antibiotics tested. As previously observed, overexpression of PDC-1 increased the MICs of ceftazidime and cefepime while having minimal impact on the efficacy of ceftazidime/avibactam, ceftolozane/tazobactam, and cefiderocol. Compared to PDC-1, most of the studied variants displayed similar cephalosporin resistance phenotypes, characterized by a pronounced effect on ceftolozane/tazobactam MICs, without significantly affecting cefiderocol resistance, as confirmed in both the PAO1 and PAO Δ*piuC* genetic backgrounds. On the other hand, production in PAO1 of the Ω-loop variants ∆P215-G222, E219K, and the dual F121L + E219K substitution had a significant impact on ceftolozane/tazobactam MICs and, particularly, on cefiderocol MICs (2–4 mg/L; four to five twofold dilution increase). The L293P variant, the unique change falling within helix H10, revealed a distinct phenotype, with the greatest impact on cefepime and cefiderocol MICs (4 mg/L, five twofold dilution increase), while having negligible effects on ceftolozane/tazobactam resistance. In the PAO Δ*piuC* host strain, production of the ∆P215-G222, E219K, F121L + E219K, and L293P variants resulted in cefiderocol MICs of 32–64 mg/L, confirming their significant contribution to cefiderocol resistance.

**TABLE 1:** Antibiotic susceptibility data of the PAO1-derived recombinant isolates producing different PDC variants previously involved in ceftazidime/avibactam, ceftolozane/tazobactam, or cefiderocol resistance in *P. aeruginosa* and antibiotic susceptibility data for the PAO Δ*piuC*-derived recombinant isolates against cefiderocol (adapted from González-Pinto *et al*. [17])

	PDC amino acid variations^*a*^^,*b*^			MIC (mg/L)^*e*^^,*f*^						
				PAO1 wild type					PAO Δ *piuC*	
Strain	SANC^*c*^	Precursor numbering^*d*^	Location	CAZ (R > 8)	C/A (R > 8)	C/T (R > 4)	FEP (R > 8)	FDC (R > 2)	FDC (R > 2)	
PAO1	–	–	–	≤1	≤1	≤0.5	2	0.125	2	
PAO1 + *bla*_PDC-1 (WT)_	–	–	–	32	2	1	16	0.25	4	
PAO1 + *bla*_PDC T70I_	T70I	T96I	Helix H2	64	8	32	16	0.5	8	
PAO1 + *bla*_PDC F121L_	F121L	F147L	α3–α4 loop	64	4	16	16	0.5	8	
PAO1 + *bla*_PDC P153L_	P153L	P180L	Helix H5	128	4	16	32	0.5	8	
PAO1 + *bla*_PDC G156D_	G156D	G183D	Helix H5	64	16	32	4	0.5	8	
PAO1 + *bla*_PDC A200T_	A200T	A227T	Ω-loop	64	4	32	16	0.5	8	
PAO1 + *bla*_PDC G202S_	G202S	G229S	Ω-loop	64	2	16	16	0.5	8	
PAO1 + *bla*_PDC ∆G202-E219_	ΔG202-E219	ΔG229-E247	Ω-loop	64	16	64	8	0.125	8	
PAO1 + *bla*_PDC G214R_	G214R	G242R	Ω-loop	128	4	16	16	1	16	
PAO1 + *bla*_PDC P215L_	P215L	P243L	Ω-loop	64	4	32	8	0.5	8	
PAO1 + *bla*_PDC P215S_	P215S	P243S	Ω-loop	64	4	32	8	0.5	8	
PAO1 + *bla*_PDC ∆P215-G222_	ΔP215-G222	ΔP243-G250	Ω-loop	256	64	128	32	2	32	
PAO1 + *bla*_PDC D217N_	D217N	D245N	Ω-loop	64	2	16	16	0.5	8	
PAO1 + *bla*_PDC E219K_	E219K	E247K	Ω-loop	256	16	128	32	4	32	
PAO1 + *bla*_PDC E219G_	E219G	E247G	Ω-loop	16	≤1	8	4	0.125	8	
PAO1 + *bla*_PDC E219D_	E219D	E247D	Ω-loop	64	16	64	8	0.5	8	
PAO1 + *bla*_PDC S226ins_	S226ins	S254ins	Ω-loop	128	64	64	8	0.5	8	
PAO1 + *bla*_PDC L293P_	L293P	L320P	Helix H10	64	2	1	128	4	32	
PAO1 + *bla*_PDC F121L + E219K_	F121L + E219K	F147L + E247K	α3–α4 loop and Ω-loop	>256	128	256	32	4	64	
PAO1 + *bla*_PDC F121L + G220S_	F121L + G220S	F147L + G248S	α3–α4 loop and Ω-loop	64	2	8	32	0.25	8	

^*a*^ Previously described polymorphisms with limited impact on the enzyme substrate profile (e.g., G1D, R53Q, A71V, T79A, L173I, V178L, P247Q, S279A, I298V, V329I, and G364A) are not indicated (16, 18, 19).

^*b*^ –, No data.

^*c*^ Structural alignment-based numbering of class C β-lactamases (SANC) according to reference 20.

^*d*^ Precursor numbering considers the signal peptide of the PDC.

^*e*^ C/A, ceftazidime/avibactam; C/T, ceftolozane/tazobactam; FEP, cefepime; FDC: cefiderocol.

^*f*^ EUCAST v.15.0 breakpoints indicated.

### Impact of E219K and L293P amino acid replacements on steady-state kinetics of the PDC β-lactamase against cephalosporins

Phenotypic analysis of the recombinant strains enabled us to identify the E219K and L293P as candidate variants for kinetic characterization, based on their significant impact on cefiderocol resistance, their different β-lactam substrate profile, and their location within the PDC amino acid backbone. Thus, to investigate the biochemical mechanisms underlying resistance to cefiderocol and comparator cephalosporins in the E219K and L293P variants, these β-lactamase proteins were purified in parallel with the parental PDC-1 enzyme, and their kinetic parameters were determined. Comparative values for *K*_m_, *k*_cat_, and *k*_cat_/*K*_m_, along with their relative changes compared to PDC-1, are presented in Table 2. The PDC-1 β-lactamase kinetic parameters confirmed its limited hydrolytic activity against all cephalosporins tested, with particularly low efficiency against ceftolozane (*k*_cat_/*K*_m_ = 0.00013 µM ^-1^ s^−1^) and cefiderocol (*k*_cat_/*K*_m_ = 0.00004 µM^−1^ s^−1^). The E219K variant demonstrated enhanced cephalosporinase activity compared to PDC-1, leading to a ≈123-fold, ≈24-fold, and ≈16-fold increase in catalytic efficiency for ceftolozane, cefiderocol, and ceftazidime, respectively. This effect is mainly driven by a substantial increase in *k*_cat_ values: approximately ≈58-fold for ceftolozane (from 0.0715 to 4.18 s^−1^), ≈125-fold for cefiderocol (from 0.0036 to 0.45 s^−1^), and ≈267-fold for ceftazidime (from 0.0082 to 2.19 s^−1^). For ceftolozane, the increased catalytic constant was further supported by an improved *K*_m_, resulting in a net *k*_cat_/*K*_m_ of 0.016 µM^−1^ s^−1^. By contrast, for cefiderocol, the increase in *k*_cat_ was partly offset by a marked reduction in substrate binding affinity (*K*_m_), from 85.29 μM to 465.64 µM.

**TABLE 2:** Kinetic parameters (mean value ± standard deviation, when applied) of the wild-type PDC-1 and the PDC E219K and PDC L293P variants for nitrocefin, ceftazidime, ceftolozane, cefepime, and cefiderocol

	PDC-1 (WT)				PDC E219K				PDC L293P			
	*K*_m_ (μM)	*k*_cat_ (s^−1^)	*k*_cat_/*K*_m_ (μM^−1^ s^−1^)	Ratio	*K*_m_ (μM)	*k*_cat_ (s^−1^)	*k*_cat_/*K*_m_ (μM^−1^ s^−1^)	Ratio for PDC E219K/PDC-1	*K*_m_ (μM)	*k*_cat_ (s^−1^)	*k*_cat_/*K*_m_ (μM^−1^ s^−1^)	Ratio for PDC L293P/PDC-1
Nitrocefin	13.98 ± 0.80	21.89 ± 6.50	1.55 ± 0.38	1	24.12 ± 4.01	4.41 ± 0.18	0.19 ± 0.04	0.12	6.71 ± 1.28	18.77 ± 4.37	2.79 ± 0.12	1.80
Ceftazidime	18.08 ± 0.88	0.0082 ± 0.0007	0.00045	1	291.63 ± 27.47	2.19 ± 0.17	0.007	15.56	2.84 ± 0.31	0.0110 ± 0.0003	0.0039	8.67
Ceftolozane	554.08 ± 3.00	0.0715 ± 0.0006	0.00013	1	266.44 ± 43.54	4.18 ± 0.19	0.016	123.08	107.97 ± 28.81	0.051 ± 0.007	0.00048	3.69
Cefepime	132.06 ± 23.69	0.138 ± 0.009	0.00105	1	92.54 ± 26.29	0.25 ± 0.06	0.0027	2.57	4.58 ± 0.42	0.189 ± 0.007	0.04125	39.29
Cefiderocol	85.29 ± 9.48	0.0036 ± 0.0007	0.00004	1	465.64 ± 120.34	0.45 ± 0.03	0.00096	24.00	2.69 ± 0.10	0.0036 ± 0.0001	0.00135	33.75

The L293P variant exhibited significant differences in the kinetic mechanism relative to the E219K variant. The best substrates for this variant were cefepime (*k*_cat_/*K*_m_ = 0.04125 µM^−1^ s^−1^, 39.29-fold increase relative to PDC-1), ceftazidime (*k*_cat_/*K*_m_ = 0.0039 µM^−1^ s^−1^, 8.67-fold increase), and cefiderocol (*k*_cat_/*K*_m_ = 0.00135 µM^−1^ s^−1^, 33.75-fold increase). Unlike the E219K variant, the enhanced catalytic efficiency observed in L293P toward these substrates was primarily driven by a drastic reduction in the *K*_m_ parameter. The improvement in substrate binding affinity was particularly pronounced for cefiderocol and cefepime, with *K*_m_ values decreasing from 85.29 µM and 132.06 µM to 2.69 µM and 4.58 µM, representing ≈32-fold and ≈29-fold improvements, respectively. Consistent with MIC values, the substrate least affected by this variant was ceftolozane.

### Effects of E219K and L293P amino acid replacements on the Ω-loop plasticity and the helix H10 folding of the PDC β-lactamase

**Fig 1:**
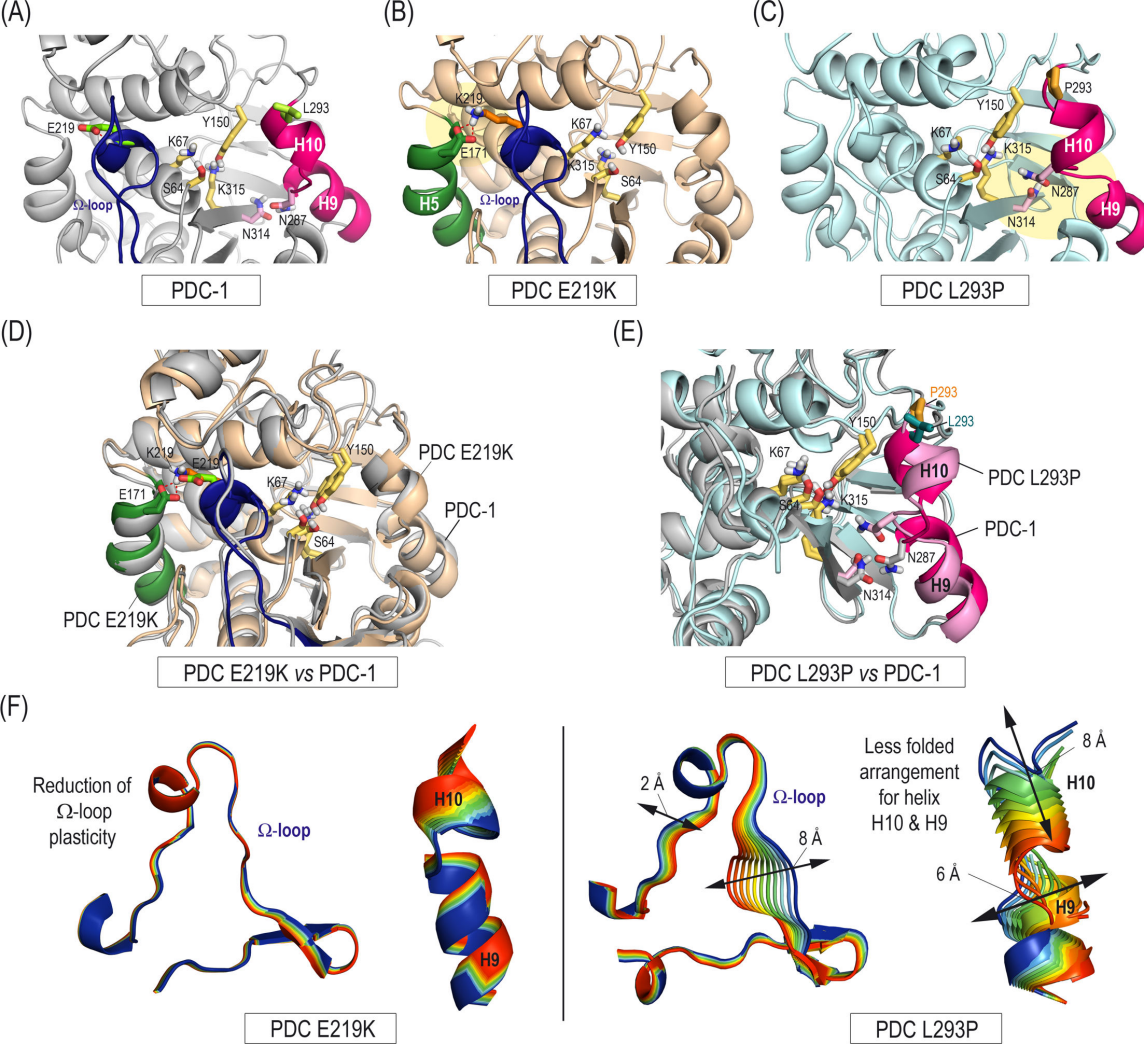
Impact of the amino acid alterations E219K and L293P on the wild-type conformation of the PDC enzymes. (A–C) Three-dimensional structure of the wild-type PDC-1 (**A**) and the E219K (**B**) and L293P (**C**) variants obtained by molecular dynamics simulation studies. Snapshots taken after 120, 100, and 200 ns of simulation, respectively. The positions of key residues involving the SVSK and KTG motifs (yellow), Ω-loop (blue), and helices H9 and H10 (magenta) are also shown. Note how the E219K alteration would promote an electrostatic interaction (yellow shadow) between residues K219 and E171 (helix H5, green). By contrast, L293P replacement would induce changes in the folding of the helix H10 (yellow shadow). (D and E) Superimposition of E219K and the L293P variants with PDC-1 highlighting the differences identified. (**F**) Overall view of the intrinsic shape-changing motions of the Ω-loop and helix H10 of the PDC E219K and PDC L293P enzymes obtained by examination of the vibrational modes. The main vibrational modes are shown. Note how the E219K change would cause a dramatic reduction in the plasticity of the Ω-loop adopting a well-defined conformation. By contrast, the L293P change would enhance the ability of helix H10 (and the loop connecting helices H9 and H10) to adopt a more open conformation, thus increasing the pocket surrounded by the inner part of helix H10.

The E219K and L293P enzyme models obtained by manually replacing residues E219 and L293 by lysine and proline, respectively, in the wild-type PDC-1 crystal structure (PDB ID 4GZB [21], 1.79 Å) were subjected to 100–200 ns of dynamic simulation using our previously reported protocol (22). The parent enzyme PDC-1 was also used as control (Fig. 1A through C). This approach yielded stable, reliable models, as indicated by the low root mean square deviation (rmsd) values obtained (Fig. S2). For the PDC-1 enzyme, the *in silico* studies revealed that the carboxylate group of residue E219 would establish an intramolecular hydrogen bond with its main NH group (Fig. 1A). In the E219K variant, this residue substitution promoted a strong electrostatic interaction between the carboxylate group of residue E171, located on helix H5, and the ε-amino group of residue K219 (Fig. 1B). This interaction markedly reduced the intrinsic plasticity of the Ω-loop, a key structural element involved in recognizing the R^1^ group of cephalosporins, also promoting a great deal of conformational freedom in the catalytic tyrosine side chain (Y150) (Fig. 1D). Interactions in PDC-1 and in the E219K variant proved very stable as no significant changes were observed throughout the simulations (Fig. S3). By contrast, the L293P substitution would increase the flexibility of both helix H10 and the small loop connecting helices H9 and H10 (Fig. 1C). As a result, helix H10 would be less folded over the enzyme, enlarging the pocket surrounded by the inner part of this helix, which is adjacent to the binding pocket of the R^2^ group of cephalosporins (Fig. 1E). Both of these effects on the Ω-loop and helix H10 were also clearly visualized by examining the vibrational modes for the E219K and L293P variants and the parent enzyme in the unbound form, calculated by principal component analysis, as implemented in AMBER (Fig. 1F; Fig. S4).

### Comparative molecular dynamics simulation studies of the PDC enzymes in complex with cefiderocol, ceftolozane, and cefepime

**Fig 2:**
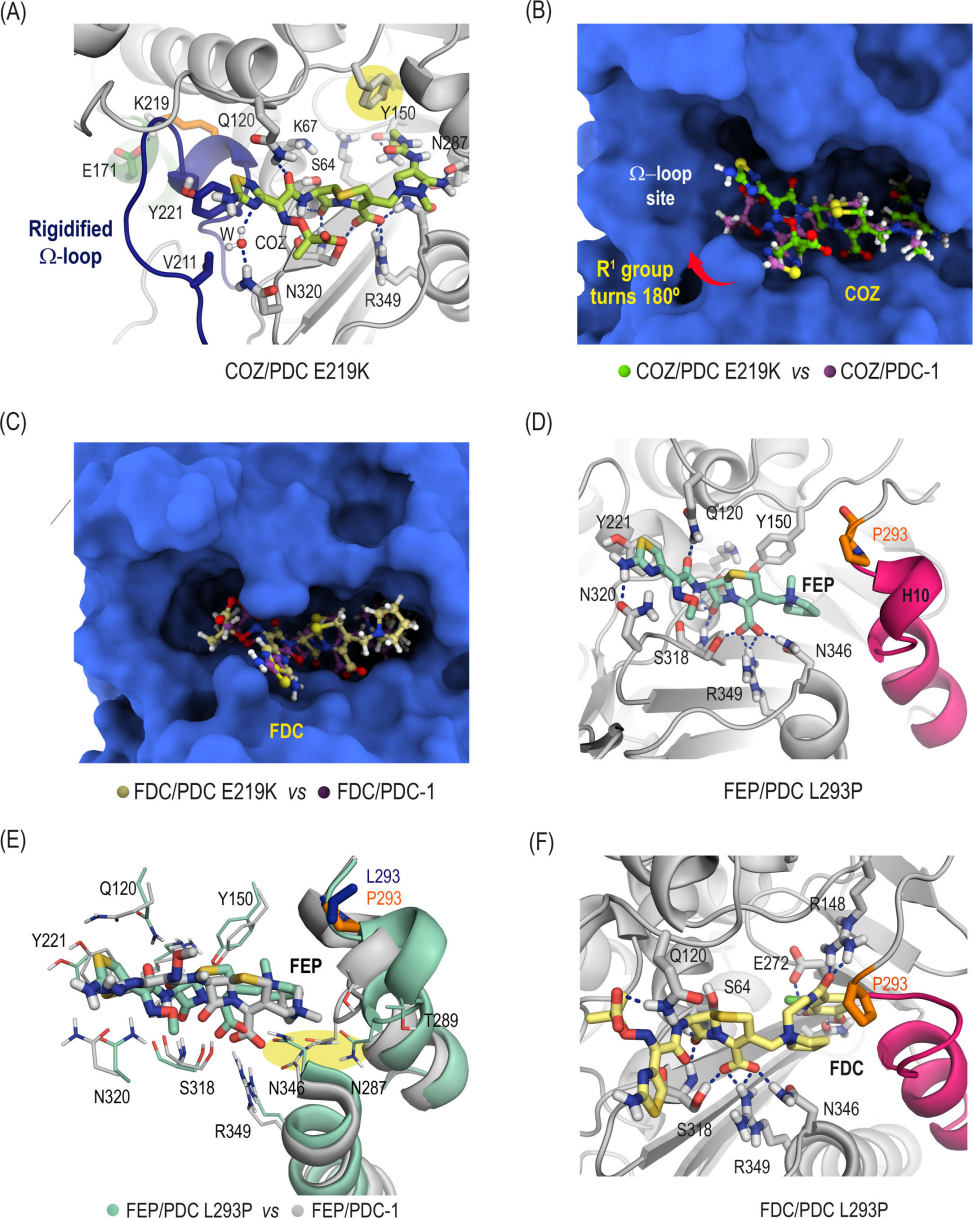
(**A and D**) Detailed views of the binding modes of ceftolozane (COZ) and cefepime (FEP) in the active site of the PDC E219K and PDC L293P variants, respectively, obtained by molecular dynamics simulation studies. Snapshots taken after 100 and 70 ns of simulation, respectively. Relevant hydrogen-bonding interactions (blue dashed lines) and key residues are shown and labeled. The amino acid alterations, K219 and P293 (orange), along with the enzyme sites undergoing relevant conformational changes, Ω-loop (blue) and helix H10 (pink), are highlighted. (**B and E**) Comparison of the binding modes of COZ and FEP in the active site of PDC E219K and PDC L293P enzymes, respectively, and the parent PDC-1 enzyme. Note how the R^1^ group in COZ would undergo a 180° turn when binding to the E219K variant, and FEP would also modify its overall binding mode against the L293P variant. (**C**) Comparison of the binding mode of cefiderocol (FDC) in the active site of PDC E219K and PDC-1 enzymes. Snapshots taken after 100 ns of simulation. (**F**) Detailed view of the binding mode of FDC in the active site of PDC L293P. Snapshot taken after 100 ns of simulation.

The Michaelis complexes of ceftolozane and cefiderocol with the E219K variant and those of cefepime and cefiderocol with the L293P variant, along with their corresponding complexes with PDC-1, were initially generated by docking using the program GOLD (23) and further analyzed in simulation studies (Fig. 2). The outcomes revealed that rigidification of the Ω-loop, induced by the E219K substitution, promotes optimal accommodation of the R^1^ group in ceftolozane. This structural adaptation embeds the aromatic and carbonyl amide groups of ceftolozane toward the enzyme groove (Fig. 2A and B), enhancing the arrangement of ceftolozane for nucleophilic attack by the catalytic serine residue, S64 (Michaelis complex). Additionally, the carbonyl group in the R^2^ moiety of ceftolozane forms a strong hydrogen bond with the amide side chain of residue Q120. This interaction, along with a set of favorable apolar contacts involving the aromatic moiety of ceftolozane, probably explains the improvement in *K*_m_ relative to PDC-1. The reduced plasticity of the Ω-loop would also improve the acylation process, which may explain the experimentally observed increase in *k*_cat_, since the β-lactam group would be located closer to the catalytic serine. The precise control of the R^1^ substituent conformation in the hydrolyzed cephalosporin (acyl-enzyme adduct) would also facilitate the efficient positioning of the deacylating water molecule, which would approach the carbonyl group from the side opposite the R^1^ group, thus enabling the release of the inactivated cephalosporin, as observed in the structural analysis of PDB ID 1IEL (2.0 Å) (24). For the cefiderocol/PDC E219K complex, while the R^1^ group of cefiderocol would adopt a similar arrangement to that observed in PDC-1, the same is not true for the R^2^ group, particularly its pyrrolidinium moiety (Fig. 2C). This group would be less deeply buried in the pocket formed by the inner part of helix H10, therefore resulting in weaker overall binding interactions with the active site of the E219K variant. This observation suggests that the rigidity of the Ω-loop, to some extent, penalizes the affinity of cefiderocol for the E219K enzyme, which is consistent with the experimentally observed increase in *K*_m_. Notably, among the cephalosporins studied, cefiderocol and ceftolozane possess the largest R^2^ substituents, although with distinct architectures (Fig. S1A). Nonetheless, as both cephalosporins form structurally similar acyl-enzyme adducts (Fig. S1B), the rigidity of the Ω-loop similarly facilitates more efficient hydrolysis of the adduct compared to PDC-1, contributing to the observed increase in *k*_cat_/*K*_m_.

On the other hand, our simulation studies revealed that the increased flexibility of the helix H10, together with the small loop connecting it to the helix H9 observed in the L293P variant, would significantly impact the binding mode of cefepime, the cephalosporin with the smallest R^2^ chain (Fig. 2D and E; Fig. S1A). Consequently, both the R^1^ and R^2^ groups in cefepime would be in closer contact and more deeply buried within the cleft of the active site and adjacent grooves, potentially explaining the ≈29-fold increase in *K*_m_ (Fig. S5). Furthermore, while the overall binding conformation of cefiderocol against the L293P and the wild-type variant would be quite similar, hydrogen-bonding interactions involving the R^2^ group within the pocket surrounded by the inner part of the helix H10 would be stronger in L293P, mainly due to the proximity and optimal arrangement of key residues (Fig. 2F; Fig. S6). Specifically, in the cefiderocol/PDC L293P complex, the R^2^ group in cefiderocol is stabilized by three strong hydrogen-bonding interactions with residues N314, E272, and R148, which proved to be very stable during the simulation (Fig. S6). Among these, the double hydrogen-bonding interaction involving the side chain carbonyl group of cefiderocol and the guanidium group of R148 is of particular importance for the cefiderocol/PDC L293P complex. The cephalosporin/PDC complexes analyzed here proved highly stable, as revealed by rmsd analysis of the enzymes and ligands throughout the simulations (Fig. S7 and S8).

## DISCUSSION

This study aimed to analyze the relative stability of cefiderocol and other cephalosporins against different, clinically encountered PDC variant enzymes, with particular focus on the biochemical and structural effects conferred by the E219K and L293P amino acid substitutions. As previously observed, the results with isogenic strains confirmed that many PDC amino acid substitutions which confer resistance to ceftolozane/tazobactam and/or ceftazidime/avibactam had a limited impact on cefiderocol resistance (17). These findings highlight the stability of cefiderocol against both wild-type PDC in *P. aeruginosa* and most derivatives that have become recalcitrant to these combinations. These observations reinforce previous findings with clinical strains showing that cefiderocol MIC values generally remain below 2 mg/L, even in *P. aeruginosa* isolates overproducing these variants (25).

Conversely, analysis of the Ω-loop alterations ∆P215-G222, E219K, and the dual F121L + E219K substitution in the PDC revealed that these variants confer resistance to both ceftolozane/tazobactam and ceftazidime/avibactam, but also significant cross-effects in cefiderocol resistance. This is concerning, given that the E219K variant is one of the most frequent substitutions observed in *P. aeruginosa* strains recovered from patients who have received therapy with ceftolozane/tazobactam or ceftazidime/avibactam (26). Although *P. aeruginosa* mutants harboring E219K often remain susceptible to cefiderocol, they usually exhibit a notable MIC increase relative to the baseline isolate (27). On the other hand, analysis of the phenotypic effect of the L293P substitution, which is located within helix H10, demonstrated that this variant confers increased resistance to cefepime and cefiderocol while maintaining susceptibility to cephalosporin/β-lactamase inhibitor combinations. This mutation has been obtained following *in vitro* cefiderocol exposure in genetically unrelated *P. aeruginosa* strains, highlighting that this amino acid replacement is under strong positive selection (28). Furthermore, L293P has also been identified in AmpC-producing Enterobacterales species previously exposed to cefepime (29), alarmingly suggesting that the use of cefepime could select for cefiderocol resistance and vice versa. Furthermore, our experimental model adds further evidence about how cefiderocol resistance may increase to higher levels (MIC ≥32 mg/L) if E219K or L293P substitutions are combined with inactivating mutations affecting iron uptake (e.g., ∆*piuC*).

Kinetic and structural analyses of PDC-1 and its E219K and L293P variants provided additional insights into the evolution of PDC-mediated resistance from classical (ceftazidime and cefepime) to newer cephalosporins (ceftolozane and cefiderocol) in *P. aeruginosa*. As previously noted, PDC-1 β-lactamase displays limited efficiency in hydrolyzing most cephalosporins, but particularly ceftolozane and cefiderocol, highlighting their increased stability (30, 31). Our findings also reinforce previous observations about how the E219K substitution significantly accelerates cephalosporin hydrolysis by increasing turnover rates (*k*_cat_), which ultimately increase the *k*_cat_/*K*_m_ values toward ceftazidime and particularly for ceftolozane, confirmed as the preferred substrate for this variant. Similar effects have been reported for other Ω-loop variants (30, 32, 33). On the other hand, to our knowledge, this is the first detailed investigation of the impact of the E219K variant on cefiderocol hydrolysis. Our data show that E219K accelerates cefiderocol hydrolysis through a very large increase in *k*_cat_ values, although this is partly mitigated by a collateral loss of binding affinity (increase in *K*_m_). In this regard, other previous structural observations with this variant led to the conclusion that E219K induces a significant expansion of the active site, particularly at the R^1^ site, enabling the accommodation of bulkier substrates. These structural changes have also been associated with significant effects on protein flexibility and stability (30, 33, 34). Conversely, our detailed examination of the underlying structural mechanisms revealed that this catalytic behavior is induced by the more constrained conformation of the Ω-loop, which ultimately facilitates the correct arrangement of ceftolozane and, to a lesser extent, cefiderocol, for nucleophilic attack by the catalytic serine residue. These findings are consistent with the recent observations of Chen *et al.*, who reported reduced root mean square fluctuations in the Ω-loop region of the E219K variant relative to the wild-type variant PDC-3, suggesting that this region is more conformationally restricted in the E219K variant (35).

This work also confirms and expands current knowledge regarding the impact of the L293P change on cefiderocol resistance. Notably, this role in cefepime resistance was characterized more than two decades ago through site-directed mutagenesis and kinetic analysis using the chromosomal AmpC of *Enterobacter cloacae* as a model (36). In line with our observations, previous findings suggest that the underlying kinetic mechanism of this phenotype can be attributed to a significant reduction in *K*_m_ values, which ultimately resulted in a ≈28-fold increase in catalytic efficiency toward cefepime. Although cefiderocol was not clinically available at that time, the major role and cross-resistance effects of mutations in the R^2^ region of AmpC in resistance to cefepime and cefiderocol have recently been confirmed by Shields *et al.*, who identified a 2-amino acid deletion (ΔA292-L293) affecting the L293 residue of the *Enterobacter hormaechei* AmpC β-lactamase, isolated from a patient treated with cefepime (37). Later, Kawai *et al*. characterized another *E. cloacae* AmpC variant with a ∆A294-P295 deletion, whose biochemical analysis revealed a ≈19-fold increase in catalytic efficiency toward cefiderocol relative to the parent enzyme (38), as we observed for the PDC L293P variant. Thus, we add further evidence of the effect of local arrangement in the R^2^ loop on the improved substrate binding of cephalosporins, including cefiderocol, in class C β-lactamases.

Altogether, our findings conclusively demonstrate that the PDC β-lactamase may accelerate cefiderocol hydrolysis by accommodating E219K and L293P amino acid substitutions, which respectively affect the plasticity of the Ω-loop and folding of the helix H10, thus exploiting the structural similarities with other cephalosporins. This study also brings attention to the interplay and cross-effects between the use of classical (ceftazidime and cefepime) and newer (ceftolozane and cefiderocol) antipseudomonal cephalosporins and the evolution of AmpC-mediated resistance. Our research therefore raises important questions that should be considered when combating *P. aeruginosa* infections, in order to expand the lifespan of cefiderocol. These observations highlight the complexity of AmpC-mediated resistance in *P. aeruginosa* and emphasize the need for ongoing active surveillance during treatment of these infections.

## MATERIALS AND METHODS

### Recombinant isolates

We took advantage of a previously constructed and characterized laboratory collection of recombinant *P. aeruginosa* PAO1 isolates expressing different *bla*_PDC_ variants, but specifically enriched for this work with additional variants, following the previously described methodology (17). The PAO Δ*piuC* strain, a PAO1 transposon mutant for the *piuC* gene (39), which exhibits impaired iron uptake and yields a baseline cefiderocol MIC of 2 mg/L, was also used as host to obtain a more detailed perspective of the different variants on cefiderocol resistance. The final collection comprised 40 recombinant isolates (20 in PAO1 and 20 in PAO Δ*piuC*) producing the wild-type PDC-1 variant from PAO1 and 19 derivatives carrying specific amino acid substitutions previously associated with the development of resistance to ceftazidime/avibactam, ceftolozane/tazobactam, or cefiderocol: T70I, F121L, P153L, G156D, A200T, G202S, ΔG202-E219, G214R, P215L, P215S, ΔP215-G222, D217N, E219K, E219G, E219D, S226ins, L293P, F121L + E219K, and F121L + G222S.

### Antimicrobial susceptibility testing

The MICs of ceftazidime, ceftazidime/avibactam, ceftolozane/tazobactam, cefepime, and cefiderocol were determined in triplicate, for all isolates, by reference broth microdilution assays. Avibactam and tazobactam were fixed at 4 mg/L. All assays were performed using cation-adjusted Mueller-Hinton broth (CAMHB), except for cefiderocol, which was determined in all cases using iron-depleted CAMHB, prepared according to CLSI M100 guidelines (40). EUCAST v.15.0 clinical breakpoints and guidelines (http://www.eucast.org/clinical_breakpoints/) were used for reference purposes. Reference strains *Escherichia coli* ATCC 25922, *Klebsiella pneumoniae* ATCC 700603, and *P. aeruginosa* ATCC 27853 were used as controls.

### Protein purification

For purification of the enzymes PDC-1, PDC E219K, and PDC L293P, the *bla*_PDC_ genes were cloned into the pGEX-6P-1 plasmid (Cytiva, Massachusetts, USA) and electroporated to the protease-deficient *E. coli* BL21 following previously described protocols (5). Protein production was induced using isopropyl β-D-1-thiogalactopyranoside (IPTG), which resulted in a fusion protein between a glutathione S-transferase (GST) and the PDC enzyme. The obtained GST-PDC fusion proteins were purified with the GST Gene Fusion System (Cytiva), following the manufacturer’s instructions. The GST moiety was thus cleaved off, and protein purity (99%) was ascertained using SDS-PAGE.

### Steady-state kinetics

The kinetic parameters of PDC-1, PDC E219K, and PDC L293P variants for ceftazidime, ceftolozane, cefepime, and cefiderocol were determined using an EPOCH 2 microplate spectrophotometer (Biotek, Vermont, USA) and a Specord 200 Plus spectrophotometer (Analytik Jena, Thuringia, Germany). The *K*_m_ (affinity, concentration of antibiotic at which the reaction rate is half of *V*_max_) values of each antibiotic were determined as *K_i app_* (affinity of the enzyme for the inhibitor/antibiotic), measuring the residual β-lactamase activity using nitrocefin as the reporter substrate. The *k*_cat_ (catalytic rate constant) values were determined by monitoring direct hydrolysis of the antibiotic at a substrate concentration much greater than *K*_m_ (at least three times the *K*_m_), to ensure the reaction was at *V*_max_ (5). The following wavelengths (*λ*) and extinction coefficients (*ε*) were used for each antibiotic: *λ* = 482 nm and *ε* = 15,900 M^−1^ cm^−1^ for nitrocefin; *λ* = 260 nm and *ε* = −8860 M^−1^ cm^−1^ for ceftazidime; *λ* = 254 nm and *ε* = −6810 M^−1^ cm^−1^ for ceftolozane; *λ* = 267 nm and *ε* = −9120 M^−1^ cm^−1^ for cefepime; and *λ* = 259 nm and *ε* = −9430 M^−1^ cm^−1^ for cefiderocol (5, 41, 42). Each parameter was determined in triplicate with 10 mM phosphate-buffered saline, pH 7.4, at room temperature.

### Building of the PDC E219K and L293P variant models

The three-dimensional structures of PDC E219K and PDC L293P were created by manual replacement of residues E219 by K219 and L293 by P293, respectively, in the available crystal structure of AmpC from *P. aeruginosa* PAO1 (PDC-1) in the wild-type form (PDB ID 4GZB [21], 1.79 Å).

### Molecular dynamics simulation studies on the PDC enzymes in the unbound form

Following our previously developed protocol (22), the PDC E219K and PDC L293P variant enzymes were immersed in a truncated octahedron of TIP3P water molecules and neutralized using the molecular mechanics force field ff14SB and GAFF of AMBER before being subjected to 100–200 ns of dynamic simulation (43). The Amber20 and AmberTools21 suite of programs were used (44). The cpptraj module in Amber20 was used to analyze the trajectories and to calculate the rmsd of the PDC enzymes during the simulation. The molecular graphics programs PyMOL (45) and UCSF ChimeraX (46) were used for visualization and depiction of enzyme figures.

### Building of the Michaelis complexes by molecular docking

We used the GOLD program v.2021.3.0 (23) and the enzyme coordinates found in PDC-1, PDC E219K, and PDC L293P after 80, 85, and 190 ns of simulation, respectively. The geometry of cefiderocol, ceftolozane, and cefepime was minimized using the AM1 Hamiltonian as implemented in the program Gaussian 09 (47) and used as MOL2 files. The ligands were docked in 25 independent genetic algorithm (GA) runs, and for each of these, a maximum number of 100,000 GA operations were performed on a single population of 50 individuals. Operator weights for crossover, mutation, and migration in the entry box were used as default parameters (95, 95, and 10, respectively), as well as the hydrogen bonding (4.0 Å) and van der Waals (2.5 Å) parameters. The position of the catalytic serine residue was used to define the docking region, and the radius of the selected spherical region was set at 6 Å. All water molecules and ions were removed for docking. The “flip ring corner” flag was switched on, while all the other flags were switched off. The GOLD scoring function was used.

### Molecular dynamics simulation studies on the ligand/enzyme binary complexes

The highest score solutions of cefiderocol, ceftolozane, and cefepime, obtained by docking with the PDC-1 and variant enzymes, were used as a starting point for MD simulations of the corresponding ligand/enzyme complexes. Docking was performed as indicated for the unbound enzyme forms.

### Vibrational mode analysis

The vibrational modes for PDC enzymes were calculated by principal component analysis with the cpptraj module from the corresponding MD trajectories (48).

## SUPPLEMENTAL MATERIAL

Supplemental material aac.00292-25-s0001.docx Fig. S1 to S8.

ASM does not own the copyrights to Supplemental Material that may be linked to, or accessed through, an article. The authors have granted ASM a non-exclusive, world-wide license to publish the Supplemental Material files. Please contact the corresponding author directly for reuse.

## References

[1] López-Causapé C, Cabot G, Del Barrio-Tofiño E, Oliver A. 2018. The versatile mutational resistome of Pseudomonas aeruginosa. Front Microbiol 9:685. doi:10.3389/fmicb.2018.00685 29681898 PMC5897538

[2] Reyes J, Komarow L, Chen L, Ge L, Hanson BM, Cober E, Herc E, Alenazi T, Kaye KS, Garcia-Diaz J, et al.. 2023. Global epidemiology and clinical outcomes of carbapenem-resistant Pseudomonas aeruginosa and associated carbapenemases (POP): a prospective cohort study. Lancet Microbe 4:e159–e170. doi:10.1016/S2666-5247(22)00329-9 36774938 PMC10016089

[3] Cosentino F, Viale P, Giannella M. 2023. MDR/XDR/PDR or DTR? Which definition best fits the resistance profile of Pseudomonas aeruginosa? Curr Opin Infect Dis 36:564–571. doi:10.1097/QCO.0000000000000966 37930070 PMC10836784

[4] Cabot G, Bruchmann S, Mulet X, Zamorano L, Moyà B, Juan C, Haussler S, Oliver A. 2014. Pseudomonas aeruginosa ceftolozane-tazobactam resistance development requires multiple mutations leading to overexpression and structural modification of AmpC. Antimicrob Agents Chemother 58:3091–3099. doi:10.1128/AAC.02462-13 24637685 PMC4068469

[5] Arca-Suárez J, Vázquez-Ucha JC, Fraile-Ribot PA, Lence E, Cabot G, Martínez-Guitián M, Lasarte-Monterrubio C, Rodríguez-Iglesias M, Beceiro A, González-Bello C, Galán-Sánchez F, Oliver A, Bou G. 2020. Molecular and biochemical insights into the in vivo evolution of AmpC-mediated resistance to ceftolozane/tazobactam during treatment of an MDR Pseudomonas aeruginosa infection. J Antimicrob Chemother 75:3209–3217. doi:10.1093/jac/dkaa291 32728723

[6] Ruedas-López A, Alonso-García I, Lasarte-Monterrubio C, Guijarro-Sánchez P, Gato E, Vázquez-Ucha JC, Vallejo JA, Fraile-Ribot PA, Fernández-Pérez B, Velasco D, Gutiérrez-Urbón JM, Oviaño M, Beceiro A, González-Bello C, Oliver A, Arca-Suárez J, Bou G. 2022. Selection of AmpC β-lactamase variants and metallo-β-lactamases leading to ceftolozane/tazobactam and ceftazidime/avibactam resistance during treatment of MDR/XDR Pseudomonas aeruginosa infections. Antimicrob Agents Chemother 66:e0206721. doi:10.1128/AAC.02067-21 34930034 PMC8846482

[7] Alonso-García I, Vázquez-Ucha JC, Lasarte-Monterrubio C, González-Mayo E, Lada-Salvador P, Vela-Fernández R, Aja-Macaya P, Guijarro-Sánchez P, Rumbo-Feal S, Muíño-Andrade M, Fernández-González A, Martínez-Guitián M, Beceiro A, Rodríguez-Iglesias M, Oliver A, Arca-Suárez J, Galán-Sánchez F, Bou G. 2023. Simultaneous and divergent evolution of resistance to cephalosporin/β-lactamase inhibitor combinations and imipenem/relebactam following ceftazidime/avibactam treatment of MDR Pseudomonas aeruginosa infections. J Antimicrob Chemother 78:1195–1200. doi:10.1093/jac/dkad062 36918743

[8] Kohira N, West J, Ito A, Ito-Horiyama T, Nakamura R, Sato T, Rittenhouse S, Tsuji M, Yamano Y. 2016. In vitro antimicrobial activity of a siderophore cephalosporin, S-649266, against Enterobacteriaceae clinical isolates, including carbapenem-resistant strains. Antimicrob Agents Chemother 60:729–734. doi:10.1128/AAC.01695-15 26574013 PMC4750680

[9] Ito A, Sato T, Ota M, Takemura M, Nishikawa T, Toba S, Kohira N, Miyagawa S, Ishibashi N, Matsumoto S, Nakamura R, Tsuji M, Yamano Y. 2018. In vitro antibacterial properties of cefiderocol, a novel siderophore cephalosporin, against Gram-negative bacteria. Antimicrob Agents Chemother 62:e01454-17. doi:10.1128/AAC.01454-17 PMC5740388 29061741

[10] Sato T, Yamawaki K. 2019. Cefiderocol: discovery, chemistry, and in vivo profiles of a novel siderophore cephalosporin. Clin Infect Dis 69:S538–S543. doi:10.1093/cid/ciz826 31724047 PMC6853759

[11] Gill CM, Santini D, Nicolau DP, Aktas E, Alfouzan W, Bourassa L, Brink A, Burnham C-A, Canton R, Carmeli Y, et al.. 2024. In vitro activity of cefiderocol against a global collection of carbapenem-resistant Pseudomonas aeruginosa with a high level of carbapenemase diversity. J Antimicrob Chemother 79:412–416. doi:10.1093/jac/dkad396 38153232 PMC10832583

[12] Vuillemin X, Da Silva M, Bour M, Landon C, Plésiat P, Jeannot K. 2023. Cefiderocol activity is compromised by acquired extended-spectrum oxacillinases in Pseudomonas aeruginosa. Int J Antimicrob Agents 62:106917. doi:10.1016/j.ijantimicag.2023.106917 37429451

[13] Santerre Henriksen A, Jeannot K, Oliver A, Perry JD, Pletz MW, Stefani S, Morrissey I, Longshaw C, Willinger B, Leyssene D, et al.. 2024. In vitro activity of cefiderocol against European Pseudomonas aeruginosa and Acinetobacter spp., including isolates resistant to meropenem and recent β-lactam/β-lactamase inhibitor combinations. Microbiol Spectr 12:e0383623. doi:10.1128/spectrum.03836-23 38483164 PMC10986614

[14] Gomis-Font MA, Clari MA, López-Causapé C, Navarro D, Oliver A. 2024. Emergence of cefiderocol resistance during ceftazidime/avibactam treatment caused by a large genomic deletion, including ampD and piuCD genes, in Pseudomonas aeruginosa. Antimicrob Agents Chemother 68:e0119223. doi:10.1128/aac.01192-23 38063398 PMC10777826

[15] Pseudomonas aeruginosa derived cephalosporinase (PDC) database. Antibiotic resistance and pathogenicity of bacterial infections group – IdISBa. 2023. Available from: https://arpbigidisba.com/pseudomonas-aeruginosa-derived-cephalosporinase-pdc-database/

[16] Berrazeg M, Jeannot K, Ntsogo Enguéné VY, Broutin I, Loeffert S, Fournier D, Plésiat P. 2015. Mutations in β-lactamase AmpC increase resistance of Pseudomonas aeruginosa isolates to antipseudomonal cephalosporins. Antimicrob Agents Chemother 59:6248–6255. doi:10.1128/AAC.00825-15 26248364 PMC4576058

[17] González-Pinto L, Blanco-Martín T, Alonso-García I, Rodríguez-Pallares S, Outeda-García M, Gomis-Font MA, Fraile-Ribot PA, Vázquez-Ucha JC, González-Bello C, Beceiro A, Oliver A, Bou G, Arca-Suárez J. 2024. Impact of transferable β-lactamases and intrinsic AmpC amino acid substitutions on the activity of cefiderocol against wild-type and iron uptake-deficient mutants of Pseudomonas aeruginosa. J Antimicrob Chemother 79:3023–3028. doi:10.1093/jac/dkae326 39287983

[18] Cortes-Lara S, Barrio-Tofiño ED, López-Causapé C, Oliver A, GEMARA-SEIMC/REIPI Pseudomonas study Group. 2021. Predicting Pseudomonas aeruginosa susceptibility phenotypes from whole genome sequence resistome analysis. Clin Microbiol Infect 27:1631–1637. doi:10.1016/j.cmi.2021.05.011 34015532

[19] Fournier D, Carrière R, Bour M, Grisot E, Triponney P, Muller C, Lemoine J, Jeannot K, Plésiat P, GERPA Study Group. 2021. Mechanisms of resistance to ceftolozane/tazobactam in Pseudomonas aeruginosa: results of the GERPA multicenter study. Antimicrob Agents Chemother 65:e01117-20. doi:10.1128/AAC.01117-20 PMC7849014 33199392

[20] Mack AR, Barnes MD, Taracila MA, Hujer AM, Hujer KM, Cabot G, Feldgarden M, Haft DH, Klimke W, van den Akker F, et al.. 2020. A standard numbering scheme for class C β-lactamases. Antimicrob Agents Chemother 64:e01841-19. doi:10.1128/AAC.01841-19 31712217 PMC7038296

[21] Lahiri SD, Mangani S, Durand-Reville T, Benvenuti M, De Luca F, Sanyal G, Docquier J-D. 2013. Structural insight into potent broad-spectrum inhibition with reversible recyclization mechanism: avibactam in complex with CTX-M-15 and Pseudomonas aeruginosa AmpC β-lactamases. Antimicrob Agents Chemother 57:2496–2505. doi:10.1128/AAC.02247-12 23439634 PMC3716117

[22] Vázquez-Ucha JC, Rodríguez D, Lasarte-Monterrubio C, Lence E, Arca-Suarez J, Maneiro M, Gato E, Perez A, Martínez-Guitián M, Juan C, Oliver A, Bou G, González-Bello C, Beceiro A. 2021. 6-halopyridylmethylidene penicillin-based sulfones efficiently inactivate the natural resistance of Pseudomonas aeruginosa to β-lactam antibiotics. J Med Chem 64:6310–6328. doi:10.1021/acs.jmedchem.1c00369 33913328

[23] Jones G, Willett P, Glen RC, Leach AR, Taylor R. 1997. Development and validation of a genetic algorithm for flexible docking. J Mol Biol 267:727–748. doi:10.1006/jmbi.1996.0897 9126849

[24] Powers RA, Caselli E, Focia PJ, Prati F, Shoichet BK. 2001. Structures of ceftazidime and its transition-state analogue in complex with AmpC beta-lactamase: implications for resistance mutations and inhibitor design. Biochemistry 40:9207–9214. doi:10.1021/bi0109358 11478888

[25] Lasarte-Monterrubio C, Fraile-Ribot PA, Vázquez-Ucha JC, Cabot G, Guijarro-Sánchez P, Alonso-García I, Rumbo-Feal S, Galán-Sánchez F, Beceiro A, Arca-Suárez J, Oliver A, Bou G. 2022. Activity of cefiderocol, imipenem/relebactam, cefepime/taniborbactam and cefepime/zidebactam against ceftolozane/tazobactam- and ceftazidime/avibactam-resistant Pseudomonas aeruginosa. J Antimicrob Chemother 77:2809–2815. doi:10.1093/jac/dkac241 35904000

[26] Fraile-Ribot PA, Cabot G, Mulet X, Periañez L, Martín-Pena ML, Juan C, Pérez JL, Oliver A. 2018. Mechanisms leading to in vivo ceftolozane/tazobactam resistance development during the treatment of infections caused by MDR Pseudomonas aeruginosa. J Antimicrob Chemother 73:658–663. doi:10.1093/jac/dkx424 29149337

[27] Simner PJ, Beisken S, Bergman Y, Posch AE, Cosgrove SE, Tamma PD. 2021. Cefiderocol activity against clinical Pseudomonas aeruginosa isolates exhibiting ceftolozane-tazobactam resistance. Open Forum Infect Dis 8:ofab311. doi:10.1093/ofid/ofab311 34262990 PMC8275882

[28] Gomis-Font MA, Sastre-Femenia MÀ, Taltavull B, Cabot G, Oliver A. 2023. In vitro dynamics and mechanisms of cefiderocol resistance development in wild-type, mutator and XDR Pseudomonas aeruginosa. J Antimicrob Chemother 78:1785–1794. doi:10.1093/jac/dkad172 37253034

[29] Barnaud G, Benzerara Y, Gravisse J, Raskine L, Sanson-Le Pors MJ, Labia R, Arlet G. 2004. Selection during cefepime treatment of a new cephalosporinase variant with extended-spectrum resistance to cefepime in an Enterobacter aerogenes clinical isolate. Antimicrob Agents Chemother 48:1040–1042. doi:10.1128/AAC.48.3.1040-1042.2004 14982805 PMC353102

[30] Slater CL, Winogrodzki J, Fraile-Ribot PA, Oliver A, Khajehpour M, Mark BL. 2020. Adding insult to injury: mechanistic basis for how AmpC mutations allow Pseudomonas aeruginosa to accelerate cephalosporin hydrolysis and evade avibactam. Antimicrob Agents Chemother 64:e00894-20. doi:10.1128/AAC.00894-20 32660987 PMC7449160

[31] Ito A, Nishikawa T, Ota M, Ito-Horiyama T, Ishibashi N, Sato T, Tsuji M, Yamano Y. 2018. Stability and low induction propensity of cefiderocol against chromosomal AmpC β-lactamases of Pseudomonas aeruginosa and Enterobacter cloacae. J Antimicrob Chemother 73:3049–3052. doi:10.1093/jac/dky317 30188999 PMC6198743

[32] Mack AR, Kumar V, Taracila MA, Mojica MF, O’Shea M, Schinabeck W, Silver G, Hujer AM, Papp-Wallace KM, Chen S, Haider S, Caselli E, Prati F, van den Akker F, Bonomo RA. 2023. Natural protein engineering in the Ω-loop: the role of Y221 in ceftazidime and ceftolozane resistance in Pseudomonas-derived cephalosporinase. Antimicrob Agents Chemother 67:e0079123. doi:10.1128/aac.00791-23 37850746 PMC10648885

[33] Barnes MD, Taracila MA, Rutter JD, Bethel CR, Galdadas I, Hujer AM, Caselli E, Prati F, Dekker JP, Papp-Wallace KM, Haider S, Bonomo RA. 2018. Deciphering the evolution of cephalosporin resistance to ceftolozane-tazobactam in Pseudomonas aeruginosa. MBio 9:e02085-18. doi:10.1128/mBio.02085-18 30538183 PMC6299481

[34] Cabot G, Kim K, Mark BL, Oliver A, Khajehpour M. 2023. Biochemical insights into imipenem collateral susceptibility driven by ampC mutations conferring ceftolozane/tazobactam resistance in Pseudomonas aeruginosa. Antimicrob Agents Chemother 67:e0140922. doi:10.1128/aac.01409-22 36715512 PMC9933714

[35] Chen S, Mack AR, Hujer AM, Bethel CR, Bonomo RA, Haider S. 2024. Ω-Loop mutations control the dynamics of the active site by modulating a network of hydrogen bonds in PDC-3 β-lactamase. bioRxiv:2024.02.04.578824. doi:10.1101/2024.02.04.578824 PMC12823066 41562583

[36] Vakulenko SB, Golemi D, Geryk B, Suvorov M, Knox JR, Mobashery S, Lerner SA. 2002. Mutational replacement of leu-293 in the class C Enterobacter cloacae P99 β-lactamase confers increased MIC of cefepime . Antimicrob Agents Chemother 46:1966–1970. doi:10.1128/AAC.46.6.1966-1970.2002 12019116 PMC127218

[37] Shields RK, Iovleva A, Kline EG, Kawai A, McElheny CL, Doi Y. 2020. Clinical evolution of AmpC-mediated ceftazidime-avibactam and cefiderocol resistance in Enterobacter cloacae complex following exposure to cefepime. Clin Infect Dis 71:2713–2716. doi:10.1093/cid/ciaa355 32236408 PMC7744991

[38] Kawai A, McElheny CL, Iovleva A, Kline EG, Sluis-Cremer N, Shields RK, Doi Y. 2020. Structural basis of reduced susceptibility to ceftazidime-avibactam and cefiderocol in Enterobacter cloacae due to AmpC R2 loop deletion . Antimicrob Agents Chemother 64:e00198–20. doi:10.1128/AAC.00198-20 32284381 PMC7318025

[39] Held K, Ramage E, Jacobs M, Gallagher L, Manoil C. 2012. Sequence-verified two-allele transposon mutant library for Pseudomonas aeruginosa PAO1. J Bacteriol 194:6387–6389. doi:10.1128/JB.01479-12 22984262 PMC3497512

[40] CLSI. 2020. Performance standards for antimicrobial susceptibility testing. CLSI supplement M100. 30th ed. Clinical and Laboratory Standards Institute.

[41] Vázquez-Ucha JC, Maneiro M, Martínez-Guitián M, Buynak J, Bethel CR, Bonomo RA, Bou G, Poza M, González-Bello C, Beceiro A. 2017. Activity of the β-lactamase inhibitor LN-1-255 against carbapenem-hydrolyzing class D β-lactamases from Acinetobacter baumannii. Antimicrob Agents Chemother 61:e01172-17. doi:10.1128/AAC.01172-17 28807908 PMC5655052

[42] Ito-Horiyama T, Ishii Y, Ito A, Sato T, Nakamura R, Fukuhara N, Tsuji M, Yamano Y, Yamaguchi K, Tateda K. 2016. Stability of novel siderophore cephalosporin S-649266 against clinically relevant carbapenemases. Antimicrob Agents Chemother 60:4384–4386. doi:10.1128/AAC.03098-15 27139465 PMC4914688

[43] Case DA, Aktulga HM, Belfon K, Ben-Shalom IY, Brozell SR, Cerutti DS, Cheatham TE, Cisneros GA, Cruzeiro VWD, Darden TA, Duke RE, Giambasu G. 2021. AMBER. University of California, San Francisco

[44] Case DA, Aktulga HM, Belfon K, Cerutti DS, Cisneros GA, Cruzeiro VWD, Forouzesh N, Giese TJ, Götz AW, Gohlke H, et al.. 2023. AmberTools. J Chem Inf Model 63:6183–6191. doi:10.1021/acs.jcim.3c01153 37805934 PMC10598796

[45] DeLano WL. 2008. The PyMOL molecular graphics system. DeLano Scientific LLC. Available from: http://www.pymol.org/

[46] Meng EC, Goddard TD, Pettersen EF, Couch GS, Pearson ZJ, Morris JH, Ferrin TE. 2023. UCSF ChimeraX: tools for structure building and analysis. Protein Sci 32:e4792. doi:10.1002/pro.4792 37774136 PMC10588335

[47] Frisch MJ, Trucks GW, Schlegel HB, Scuseria GE, Robb MA, Cheeseman JR, Scalmani G, Barone V, Mennucci B, Petersson GA. 2009. Gaussian 09, Revision D.01, Gaussian, Inc.: Wallingford CT

[48] AMBER. 2015. London workshop. Introduction to principal component analysis. Available from: http://www.amber.utah.edu/AMBER-workshop/London-2015/pca/

