# Supplementary material for: Functional and structural analyses of amino acid sequence variation in PDC β-lactamase reveal different mechanistic pathways toward cefiderocol resistance in Pseudomonas aeruginosa
Source: Antimicrob Agents Chemother. 2025 May 27;69(7):e00292-25. doi: 10.1128/aac.00292-25 (PMC12217463; doi:10.1128/aac.00292-25)
Supplement: Supplemental material — Fig. S1 to S8. [file aac.00292-25-s0001.docx]

Supplemental material for:

**Functional and structural analysis of amino acid sequence variation in the PDC β-lactamase reveals different mechanistic pathways towards cefiderocol resistance in *Pseudomonas aeruginosa***

**Lucía GONZÁLEZ-PINTO^1^, María Antonia GOMIS-FONT^2,3^, Emilio LENCE^4^, Michelle OUTEDA-GARCÍA^1^, Tania BLANCO-MARTÍN^1^, Salud RODRÍGUEZ-PALLARES^1^, Lucía SÁNCHEZ-PEÑA^1^, Isaac ALONSO-GARCÍA^1^, Juan Carlos VÁZQUEZ-UCHA^1,3^, Alejandro BECEIRO^1,3^, Germán BOU^1,3^, Concepción GONZÁLEZ-BELLO^4^, Antonio OLIVER^2,3 ¥ #^ and Jorge ARCA-SUÁREZ^1,3 ¥ #^**

^1^Servicio de Microbiología & Instituto de Investigación Biomédica de A Coruña (INIBIC), Complexo Hospitalario Universitario A Coruña, A Coruña, Spain

^2^Servicio de Microbiología & Instituto de Investigación Sanitaria Illes Balears (IdISBa), Hospital Universitario Son Espases, Palma de Mallorca, Spain

^3^CIBER de Enfermedades Infecciosas (CIBERINFEC), Instituto de Salud Carlos III, Madrid, Spain

^4^Centro Singular de Investigación en Química Biolóxica e Materiais Moleculares (CiQUS), Departamento de Química Orgánica, Universidade de Santiago de Compostela, Santiago de Compostela, Spain.

^¥^ Jorge Arca-Suárez and Antonio Oliver contributed equally as senior authors.

**^#^ Corresponding author: Dr. Jorge Arca Suárez**

e-mail: jorge.arca.suarez@sergas.es

Servicio de Microbiología & Instituto de Investigación Biomédica A Coruña (INIBIC)

Complexo Hospitalario Universitario A Coruña (CHUAC)

As Xubias, 15006, A Coruña, Spain.

Telephone: 0034679082502

**^#^ Alternative corresponding author: Dr. Antonio Oliver**

e-mail: antonio.oliver@ssib.es

Servicio de Microbiología & Instituto de Investigación Sanitaria Illes Balears (IdISBa)

Hospital Universitario Son Espases, Palma de Mallorca, Spain

Ctra. Valldemossa, 79, 07120 Palma de Mallorca, Spain

Telephone: 0034871205148

**Table of contents**

1. Figure S1 ……………………………………………………………………………..………………………… S3
2. Figure S2……………………………………………………………………………..………………………… S4
3. Figure S3 ……………………………………………………………………………..………………………… S5
4. Figure S4 ……………………………………………………………………………..………………………… S6
5. Figure S5 ……………………………………………………………………………..………………………… S7
6. Figure S6 ……………………………………………………………………………..………………………… S8
7. Figure S7 ……………………………………………………………………………..………………………… S9
8. Figure S8 ……………………………………………………………………………..………………………… S10

**Figure S1**. **(A)** Chemical structures of cefiderocol (FDC), ceftolozane (COZ), ceftazidime (CAZ) and cefepime (FEP). The R^2^ groups in the different cephalosporins (the main point of difference) are highlighted in blue with yellow shading. The differences in the R^1^ group involving ceftolozane and cefepime are also shown in pink and red, respectively. **(B)** General chemical structure of the enzyme adduct, i.e. after β-lactam ring opening after nucleophilic attack of the catalytic serine residue S64.


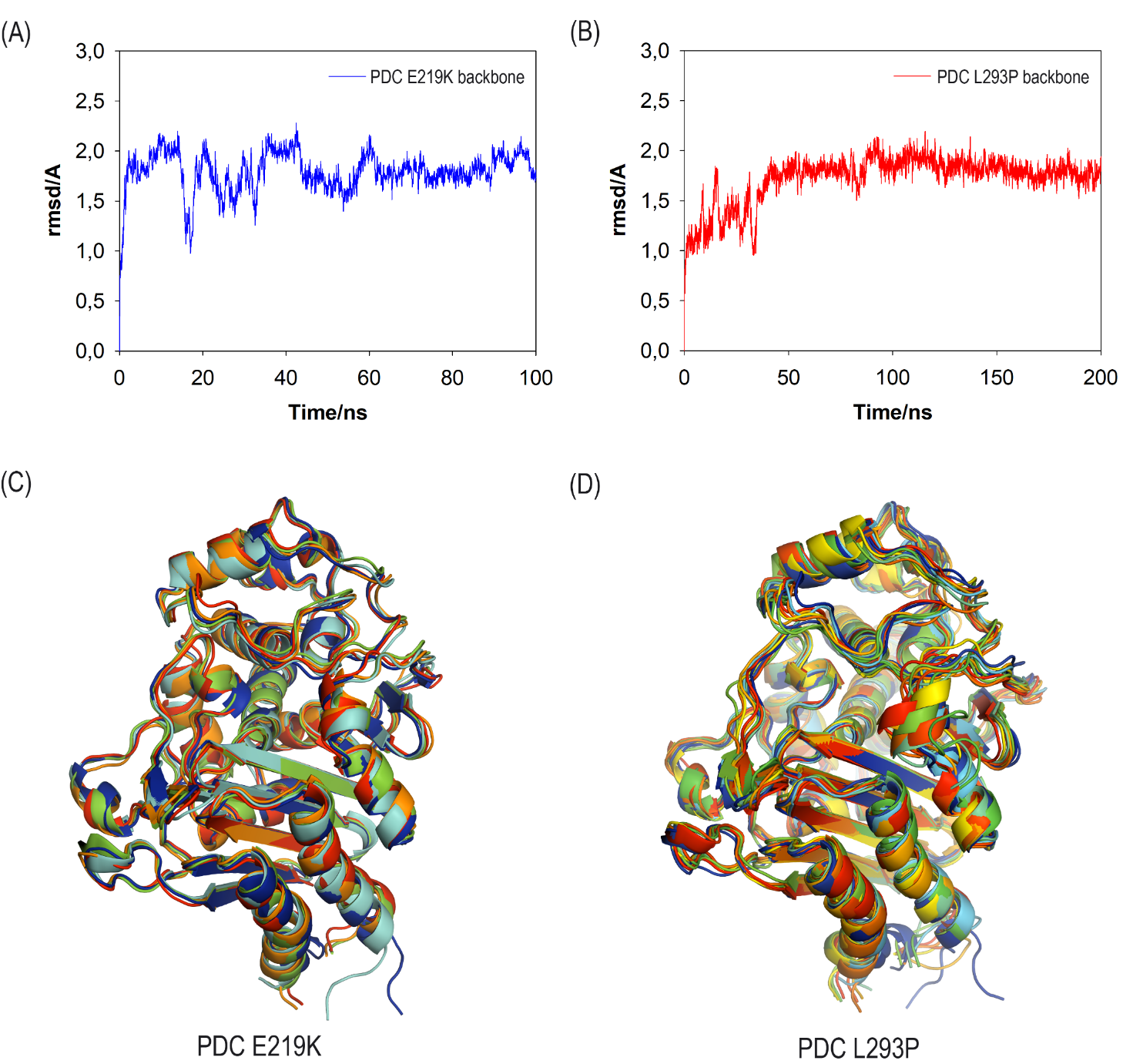


**Figure S2**. **(A, B)** Root mean square deviation (rmsd) plots for the protein backbone (Cα, C, O and N atoms) calculated from the MD simulations of enzymes: **(A)** PDC E219K; and **(B)** PDC L293P. Average low rmsd values of 1.8 and 1.7 Å, respectively, were obtained. **(C, D)** Comparison of several snapshots of the PDC E219K **(C)** and PDC L293P **(D)** wild-type forms during 100 ns and 200 ns, respectively, of dynamic simulation. The low rmsd values obtained, together with the non-relevant differences in the whole structure, reveal the high stability of both models.


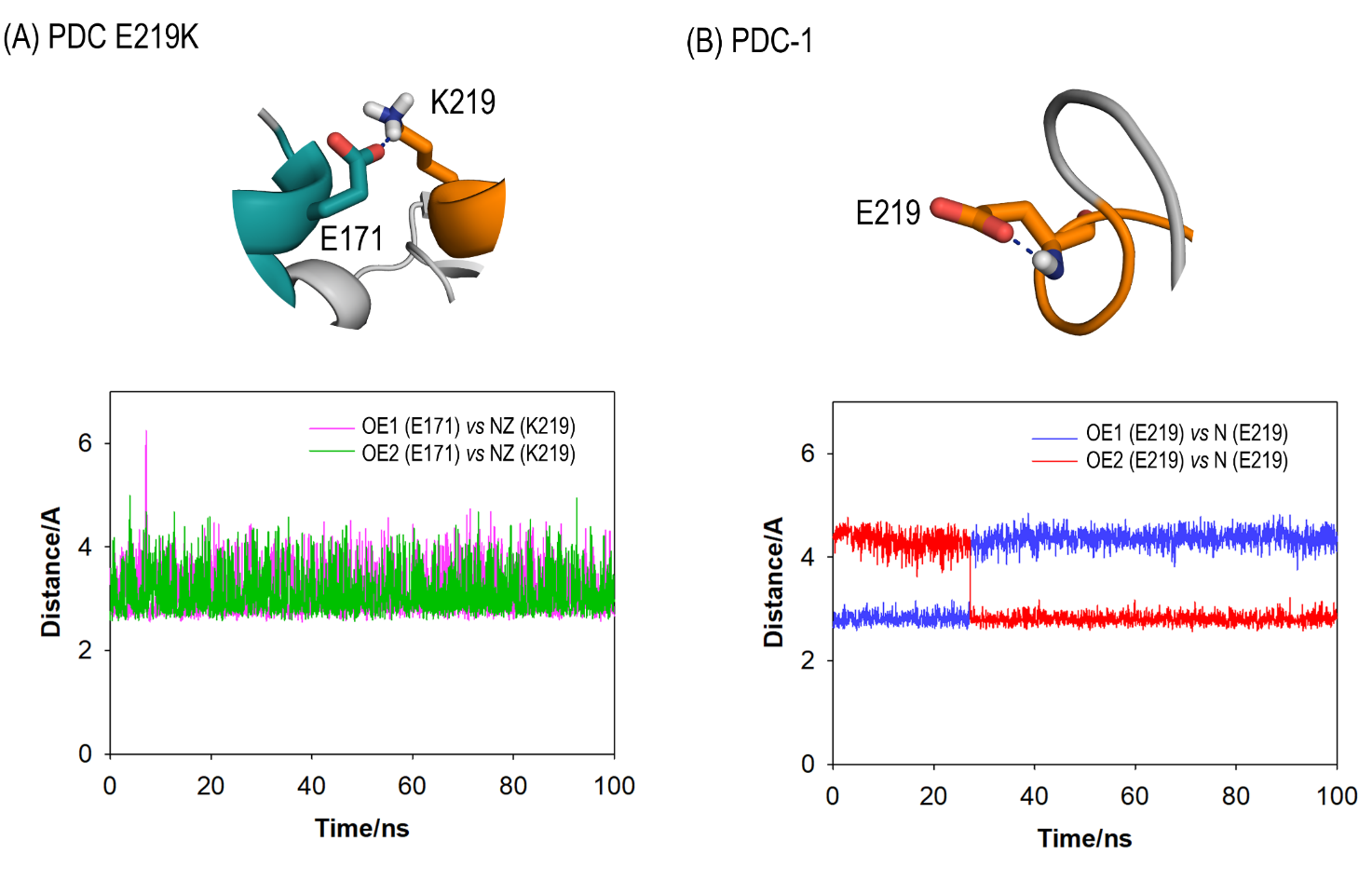


**Figure S3**. Stability of the hydrogen-bonding interaction of **(A)** the carboxylate group in residue K219 (NZ atom) with ε-amino group in residue E171 (OE1 and OE2 atoms) in PDC E219K wild-type form, and **(B)** the carboxylate group in E219 (OE1 and OE2 atoms) with its main amide group (N atom) in PDC-1 wild-type form, throughout the whole simulation. Note the high stability of both interactions.


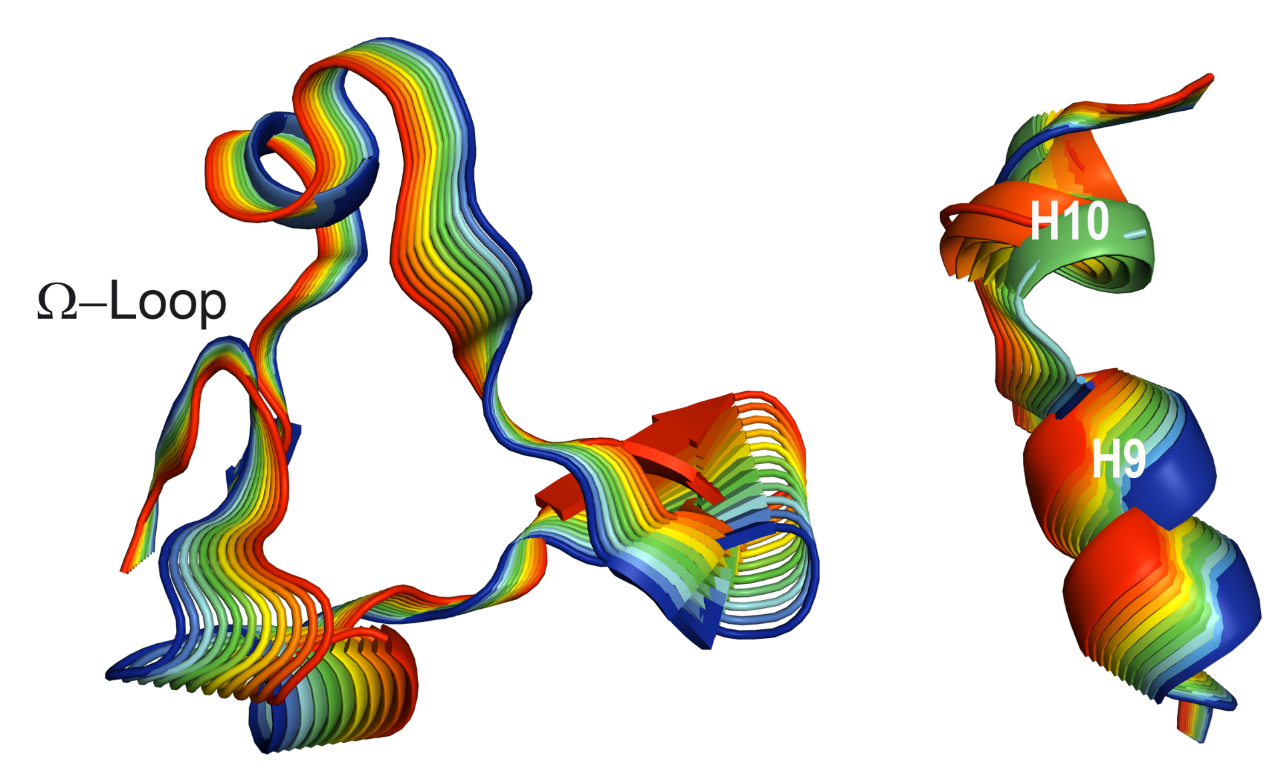


**Figure S4**. Overall view of the intrinsic shape-changing motions of the Ω-loop and the helices H9 and H10 of PDC‑1 enzyme obtained by examination of the vibrational modes. The main vibrational modes are shown. Note the high plasticity of the Ω-loop and the well-defined conformation of the helices H9 and H10.

**
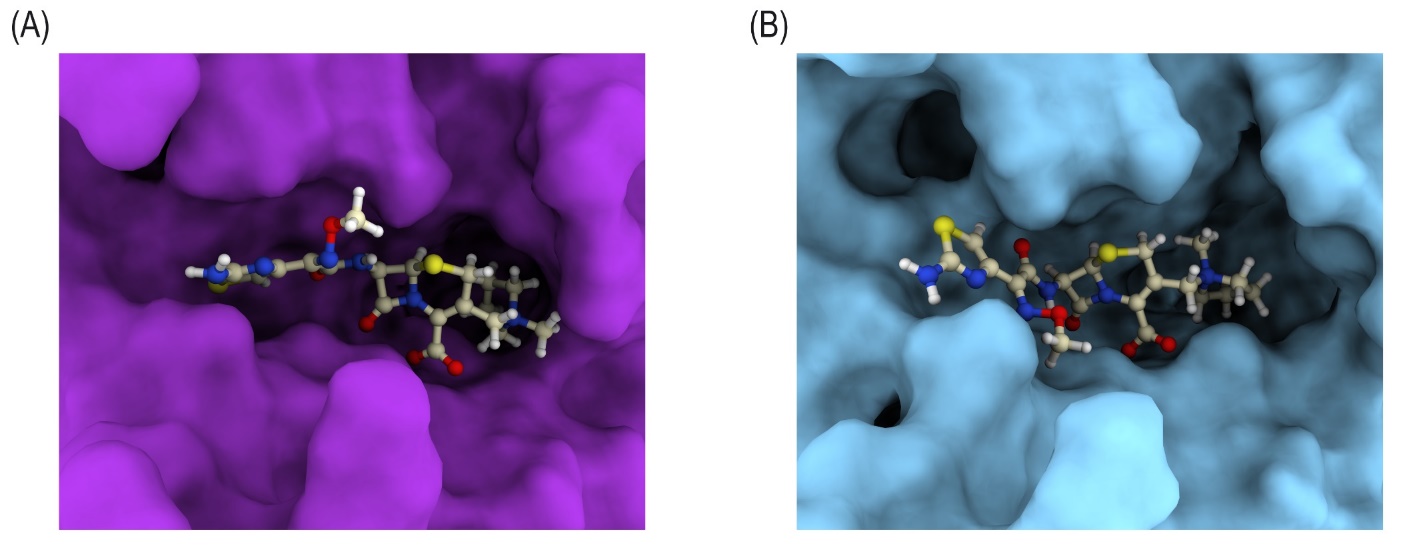
**

**Figure S5**. Overall view of the binding mode of cefepime in the active site of PDC-1 **(A)** and PDC L293P **(B)** enzymes, respectively, obtained by MD simulation studies. Snapshots taken after 100 ns and 70 ns of simulation, respectively.

**
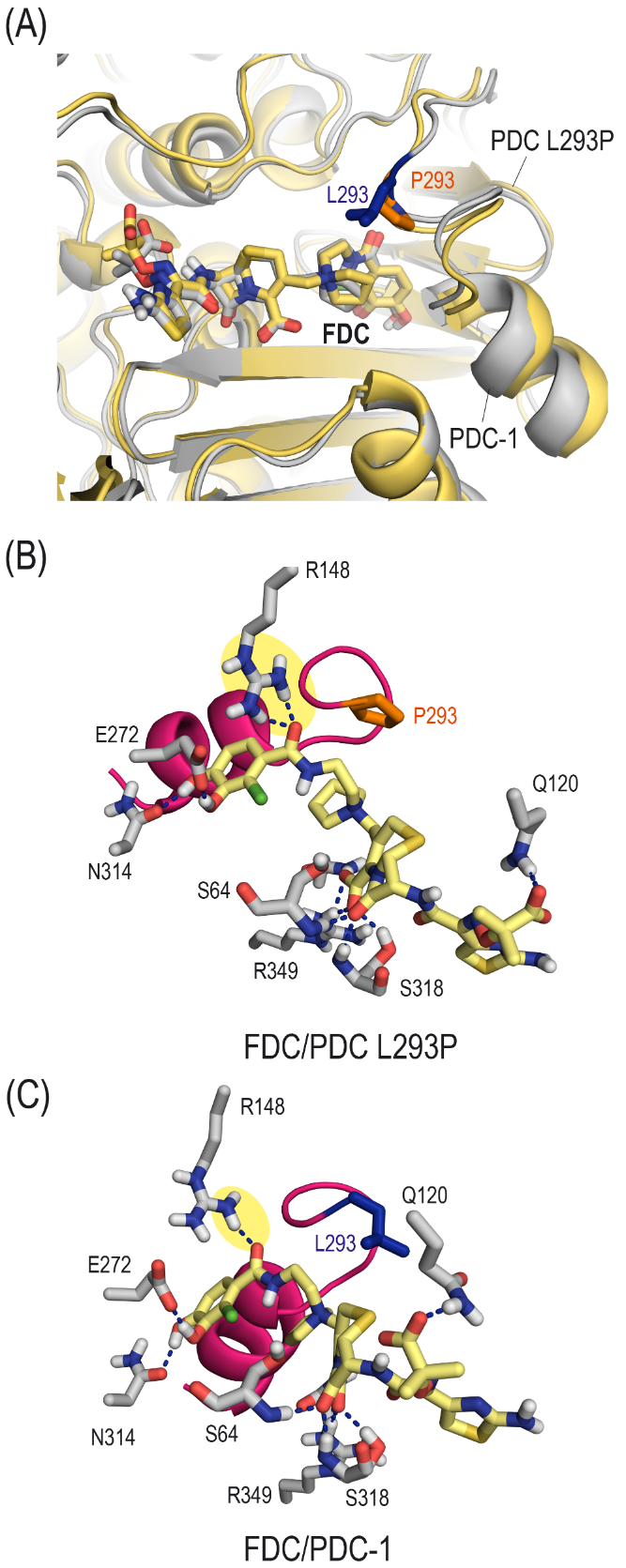
**

**Figure S6**. **(A)** Comparison of the binding mode of cefiderocol (FDC) in the active site of PDC L293P and PDC-1 enzymes. Snapshots taken after 100 ns of simulation. **(B, C)** Detailed view of the main hydrogen-bonding interactions involving the R2 and β-lactam core of FDC with PDC L293P **(B)** and PDC-1 **(C)** enzymes, respectively. Relevant hydrogen-bonding interactions (blue dashed lines) and key residues are shown and labelled. Note the stronger hydrogen-bonding interaction between residue R148 in PDC L293P and the side chain carbonyl group in FDC (yellow shadow).

**
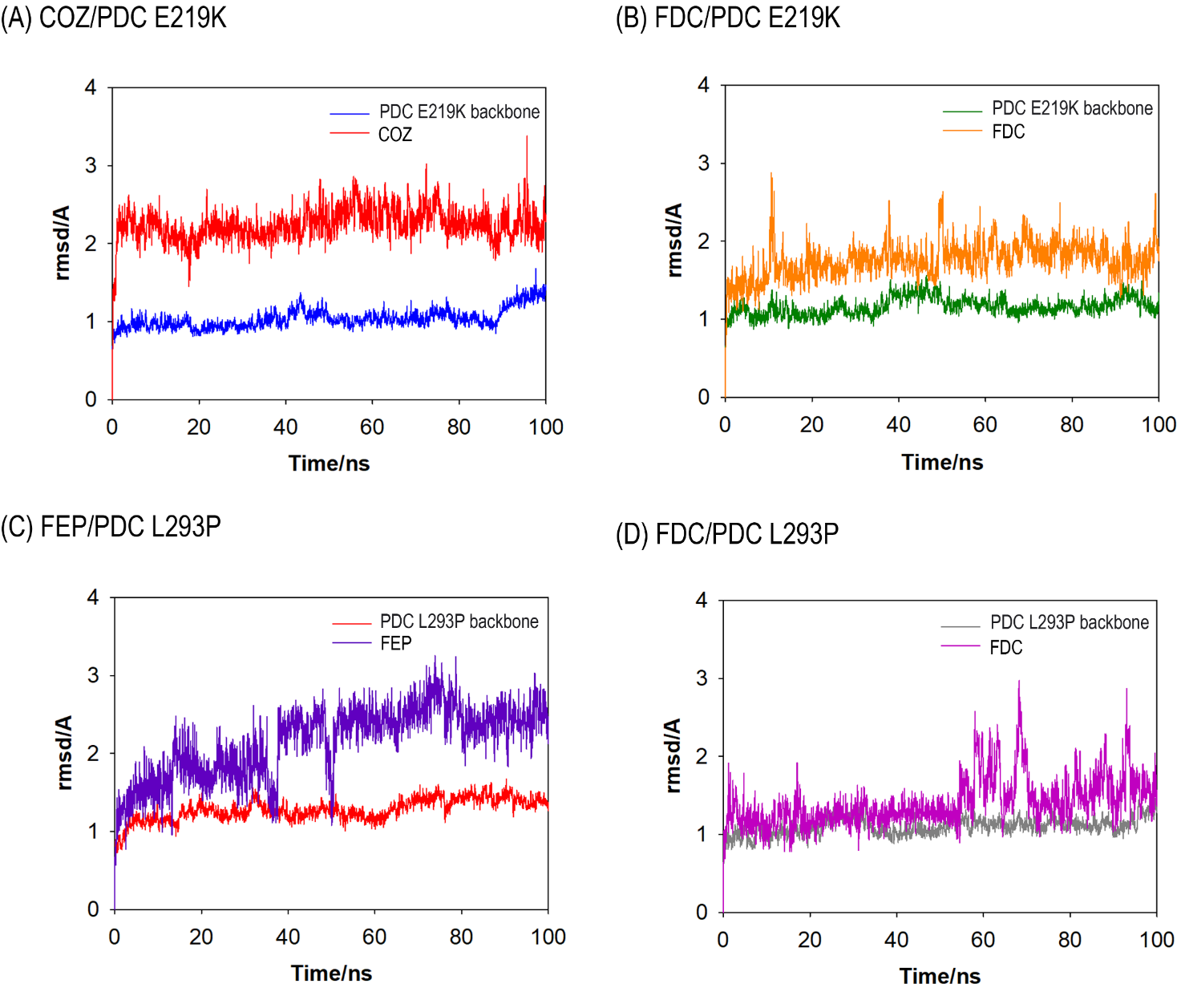
**

**Figure S7**. Root-mean-square deviation (rmsd) plots for the enzyme backbone (Cα, C, O and N atoms) and cefiderocol (FDC), ceftolozane (COZ) and cefepime (FEP), calculated from the MD simulations of enzyme complexes: **(A)** COZ/PDC E219K; **(B)** FDC/PDC E219K; **(C)** FEP/PDC L293P; and **(D)** FDC/PDC L293P. The low average rmsd values, ranging from 1.0 Å to 1.3 Å for the enzyme backbone and from 1.4 Å to 2.2 Å for the ligands obtained, together with the no relevant differences in the whole structure, reveal the high stability of the Michaelis complexes.

**
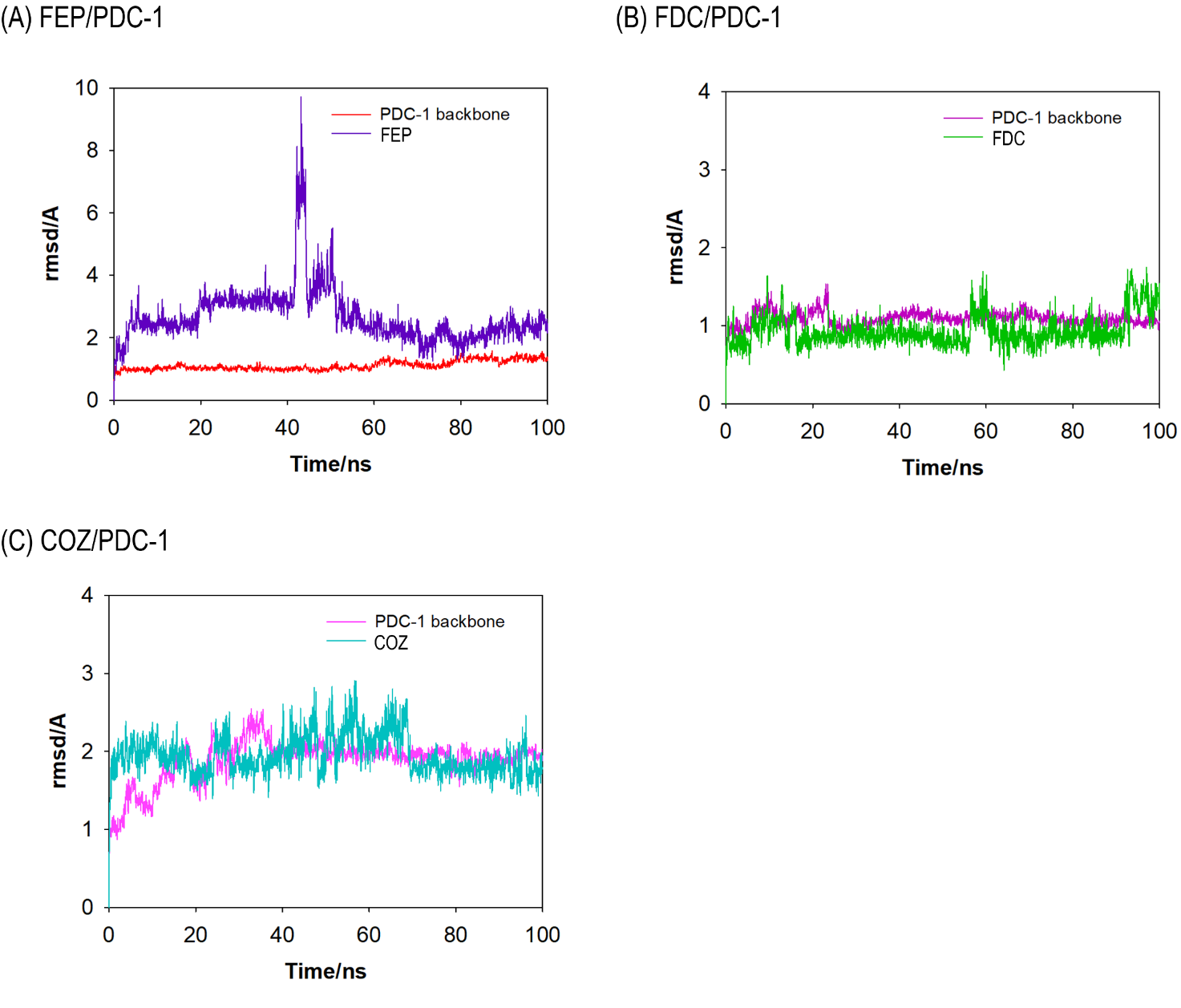
**

**Figure S8**. Root-mean-square deviation (rmsd) plots for the enzyme backbone (Cα, C, O and N atoms) and compound cefepime (FEP), cefiderocol (FDC) and ceftolozane (COZ) calculated from the MD simulations of enzyme complexes: **(A)** FEP/PDC-1; **(B)** FDC/PDC-1; and **(C)** COZ/PDC-1. The average rmsd values ranged from 1.1 Å to 1.9 Å for the enzyme backbone and from 0.9 Å to 2.7 Å for the ligands.
